# Emergency medical services key performance measurement in Asian cities

**DOI:** 10.1186/s12245-015-0062-7

**Published:** 2015-04-23

**Authors:** Nik Hisamuddin Rahman, Hideharu Tanaka, Sang Do Shin, Yih Yng Ng, Thammapad Piyasuwankul, Chih-Hao Lin, Marcus Eng Hock Ong

**Affiliations:** Department of Emergency Medicine, School of Medical Sciences, University Sains Malaysia, Kota Bharu, 16150 Malaysia; Department of EMS System, Graduate School, Kokushikan University, Tokyo, Japan; Department of Emergency Medicine, College of Medicine, Seoul National University, Seoul, Korea; Medical Department, Singapore Civil Defence Force, Singapore, Singapore; Department of Emergency Medicine, Prince of Songkla University, Hat Yai, Thailand; Department of Emergency Medicine, College of Medicine, National Cheng Kung University Hospital, National Cheng Kung University, Tainan, Taiwan; Department of Emergency Medicine, Singapore General Hospital, Singapore, Singapore

**Keywords:** EMS, Pre-hospital care, Performance, Benchmarking

## Abstract

**Background:**

One of the key principles in the recommended standards is that emergency medical service (EMS) providers should continuously monitor the quality and safety of their services. This requires service providers to implement performance monitoring using appropriate and relevant measures including key performance indicators. In Asia, EMS systems are at different developmental phases and maturity. This will create difficultly in benchmarking or assessing the quality of EMS performance across the region. An attempt was made to compare the EMS performance index based on the structure, process, and outcome analysis.

**Findings:**

The data was collected from the Pan-Asian Resuscitation Outcome Study (PAROS) data among few Asian cities, namely, Tokyo, Osaka, Singapore, Bangkok, Kuala Lumpur, Taipei, and Seoul. The parameters of inclusions were broadly divided into structure, process, and outcome measurements. The data was collected by the site investigators from each city and keyed into the electronic web-based data form which is secured strictly by username and passwords. Generally, there seems to be a more uniformity for EMS performance parameters among the more developed EMS systems. The major problem with the EMS agencies in the cities of developing countries like Bangkok and Kuala Lumpur is inadequate or unavailable data pertaining to EMS performance.

**Conclusions:**

There is non-uniformity in the EMS performance measurement across the Asian cities. This creates difficulty for EMS performance index comparison and benchmarking. Hopefully, in the future, collaborative efforts such as the PAROS networking group will further enhance the standardization in EMS performance reporting across the region.

## Findings

### Background

In today’s health care environment, the demand for objective comparative information about the performance of health care organizations and providers has created a need for data-driven evaluation processes [1]. As a part of the health care environment, pre-hospital emergency medical services (EMS) systems are no different in their need for objective comparative system information to assist government officials at all levels to establish relevant policy, select appropriate system design, and monitor system quality and effectiveness. Assessing EMS performance may be a simple task if it is carried out within a local health care system, but things can get complicated if the comparison is made between states or countries [2,3]. Different states or countries may deliver EMS differently. In Asia, EMS systems are at different developmental phases and maturity. For example, Singapore, Japan, and Korea have mature and systematic EMS whereas in other developing countries, the system is still at its infancy [4,5]. This will create difficultly benchmarking or assessing the quality of EMS performance across the region. Despite limited data, we attempted to compare basic EMS performance based on reports by the Pan-Asian Resuscitation Outcome Study (PAROS) group [6]. Hopefully, this will be the first step in understanding the quality of EMS in Asia and the stepping stone for benchmarking in the future.

#### EMS performance measurement

Measuring quality in EMS systems is challenging [7]. Measuring quality in EMS is important since EMS is the practice of medicine in the pre-hospital setting. The goal of EMS Performance Measurement (EMSPM) is to apply knowledge, data, and experience to evaluate and improve EMS service delivery, personnel performance, and clinical care. The need for increased coordination in patient care and higher quality care at lower costs has made it essential for EMS agencies to have in-place quality control or quality improvement programs that rely on key performance indicators to continuously monitor the system’s overall performance and the effectiveness of the different pre-hospital interventions [8,9]. The Institute of Medicine (IOM), in a report entitled “Emergency Medical Services at the Crossroads” and published in 2006, recommended the development of “evidence based performance indicators that can be nationally standardized so that statewide and national comparisons can be made” [10]. The development and implementation of these indicators would enhance accountability in EMS and provide EMS agencies with data to measure their system’s overall performance and to develop sound strategic quality improvement planning.

#### Performance indicators

Performance indicators are measurement tools that should be “specific, measurable, action oriented, relevant and timely” [11]. An indicator is a metric that reflects on the performance of a system or process. As the indicator value rises or falls, it suggests that the system or process is operating better or worse - like a performance thermometer. The common measurable indicators include structure, process, and outcome. (Table 1) However, the National Highway and Traffic Safety Act (NHTSA) has recommended a more comprehensive measurement which includes system design and structure, human resources (culture, training, safety, credentialing, etc.), clinical care and outcome, response, finance, quality management, and community demographics [12]. Problems may arise from a complex measurement system especially when the system is used for multiple agencies or international usage. Different agencies or countries may have different definitions for a specific measure such as response time, or the data may not be easily available within that particular community (such as pre-hospital defibrillation) [13,14]. This will create standardization of EMSPM across the region really difficult. Finding the most common available denominators is perhaps the best method to carry out the inter-agency EMSPM [15].

**Table 1:** **Performance indicators**

**Indicator type**	**Definitions**	**EMS systems performance index examples**
Structure	Characteristics of the different components of the system	(i) Facilities
		(ii) Equipment
		(iii) Staffing
		(iv) Knowledge base of providers
		(v) Credentials
		(vi) Deployment
		(vii) Response times
Process	Combination or sequence of steps in patient care intended to improve patient outcome	(i) Medical protocols
		(ii) Medication administration
		(iii) Transport to appropriate facility
Outcome	Changes in health and well-being related to antecedent care 6 Ds^a^	
	(i) Death	(i) Out of hospital cardiac arrest survival
	(ii) Disease	(ii) Patient Satisfaction
	(iii) Disability	(iii) Improvement in pain score
	(iv) Discomfort	
	(v) Dissatisfaction	
	(vi) Destitution	

^a^EMS outcomes defined by Emergency Medical Services Outcomes Project (EMSOP).

The structure of EMS agencies differs significantly between the Asian countries. The focus of emergency health care in most of the Asian countries relies heavily on the hospital system, with little emphasis on pre-hospital care other than rapid transport. The quality of care available at all levels appears to be directly related to the economic status of that country and the efforts of government and health authorities to identify and support the development of pre-hospital services. Countries like Singapore, South Korea, Japan, and Taiwan have a much more mature EMS setup compared to other countries in the Asian region [16,17]. They have robust data system collection partly for the purpose of quality improvement. Unfortunately, there are no standardized quality assurance and monitoring system within this region. Overall, comparing the EMSPM is difficult partly due to lack of data and non-standardization of the delivery system. However, we attempted to collect basic common data within the PAROS study group in 2013.

### Methods

The data collected comprises of structure, process, and outcome information of the EMS system within each of the EMS agencies. A survey form was created and sent via email to all the representatives of the EMS agencies or the medical directors of the PAROS group. Some of the survey forms were handed to the participating members by hand during the annual PAROS meetings held in various cities within Asia. Some of the data was obtained from the previous publications within the PAROS group that is adopted from cardiac arrest registry to enhance survival (CARES) study based in the USA [18,19]. Twenty-four variables were assessed and compared, all of which were derived by consensus across the PAROS countries. The components were agreed upon after series of meeting and followed the Utstein template. The most common available denominators of measurement were taken into analysis and comparison. The other variables that were not available in all the participated countries would be excluded from analysis. The overall comparison of EMSPM is carried out based on the countries and cities where the EMS agencies originate from.

### Results

The final response reported in this study is from seven cities (Seoul, Tokyo, Osaka, Taipei, Singapore, Bangkok, and Kuala Lumpur) and six countries (Singapore, South Korea, Taiwan, Japan, Thailand, and Malaysia). General demography of each of the cities is as shown in Table 2. In all, we managed to gather measurement indicators for 13 structural components, nine process components, and two outcome indicators. Korea, Singapore, Taiwan, and Japan provided the data from their existing EMS registries whereas countries like Malaysia and Thailand based their data mainly from hospitals within the big cities like Kuala Lumpur and Bangkok. Some of the data is national and others are specific only for the cities (Tables 3, 4, and 5).

**Table 2:** **General demography of the cities in the survey**

**City**	**Seoul**	**Tokyo**	**Osaka**	**Taipei**	**Singapore**	**Bangkok**	**Kuala Lumpur**
Population	10,140,000	13,185,502	2,666,371	2,652,959	5,399,200	8,280,925	1,627,172
Area (km^2^)	605.21	2,187.66	223.00	271.799	716.1	1,568.737	243
Population density per km^2^	17,000	6,000	11,759	9,586	7,540	5,300	6,891
Number of districts	25	23 wards	24 wards	12	28	50	11

**Table 3:** **Structure comparisons of EMS systems in Asia**

**City**	**Seoul**	**Tokyo**	**Osaka**	**Taipei**	**Singapore**	**Bangkok**	**Kuala Lumpur**
Operation of ambulance	Fire department	Fire department	Fire department	Fire department	Fire department	Hospital	Hospital plus NGOs
Dispatch center	Fire department	Fire department	Fire department	Fire department	Fire department	Fire department	Hospital and NGOs
Dispatcher certification	Certified	Certified	Certified	Certified	Certified	Certified	Certified
Tiered response	BLS	BLS	BLS	BLS plus ALS	BLS	BLS plus ALS	BLS or ALS
Medical direction	Direct only	Mixed	Mixed	Mixed	Indirect only	Direct only	Direct only
Dispatcher type	Firefighters	Firefighters	Firefighters	Firefighters	Firefighters	Nurse	EMD
Ambulance personnel	EMT	First aider, EMT	First aider, EMT	EMT paramedic	EMT intermediate	Nurse, EMT	Medical assistant, nurses
Ambulance station	Fire department	Fire department	Fire department	Fire department	Fire station	Hospital/clinic	Hospital/clinic
Total EMS providers	751	2,184	600	1,452	200	12	NA
Training period	>320 h for EMT-basic	750 to 1,095 h for EMT	750 to 1,095 h for EMT	1,280 h for EMT-P	2,640 h for EMT-I	6 months	120 h
Ambulance total number	117	229	72	76	40	26	60
Ambulance stations total numbers	114	229	50	43	31	2	6

**Table 4:** **Process comparison of EMS systems in Asia**

**City**	**Seoul**	**Tokyo**	**Osaka**	**Taipei**	**Singapore**	**Bangkok**	**Kuala Lumpur**
The use of AED	Yes	Yes	Yes	Yes	Yes	Yes	Yes
Mean call to arrival at scene for OHCA (min/sd)	6.8 (±3.5)	6.0 (±4.2)	7.8 (±3.6)	7.0 (±10.0)	10.2 (±4.3)	11.8 (±6.0)	22.5 (±16.0)
Mean call to arrival to hospital for OHCA (min/sd)	21.4 (±8.8)	29.0 (±9.9)	23.4 (±14.1)	15.0 (±21.0)	35.5 (±9.5)	41.8 (±21.0)	42.9 (±32.5)
Dispatcher CPR instruction	Yes	Yes	Yes	Yes	Yes	Yes	Yes
Medical oversight of dispatch	Yes	Yes	Yes	Yes	Yes	Yes	Yes
Airway management	Basic and advance	Basic and advance	Basic and advance	Basic and advance	Basic and LMA	Basic	Basic and advance
EMS-treated OHCA (%)	100%	100%	100%	100%	100%	NA	NA
Pre hospital defibrillation (%)	5.4%	8.9%	14.3%	8.7%	21.9%	NA	NA
CPR by EMS (%)	90.6%	100%	100%	90.8%	100%	NA	NA

NA, no data available.

**Table 5:** **Outcome comparison of EMS systems in Asia**

**City**	**Seoul**	**Tokyo**	**Osaka**	**Taipei**	**Singapore**	**Bangkok**	**Kuala Lumpur**
EMS ROSC	23.4%	35.1%	35%	31.1%	11.2%	9.1%	NA
Survival to admission for OHCA, *n* (%)	20.4%	27.3%	28.1%	5.9%	17.0%	27.7%	8.0%
Survival to discharge for OHCA, *n* (%)	8.9%	5.2%	7.6%	5.9%	3.2%	6.8%	1.3%
CPC	3.0%	2.8%	2.8%	3.0%	1.7%	1.7%	NA

ROSC, return of spontaneous circulation; OHCA, out-of-hospital cardiac arrest; CPC, cerebral performance score 1 or 2; NA, no available data.

### Discussion

Generally, there seems to be a more uniformity for EMS performance parameters among the more developed EMS systems. The major problem with the EMS agencies in the cities of developing countries like Bangkok and Kuala Lumpur is inadequate or unavailable of data pertaining to EMS performance. This is partly due to lack of uniform and standardized data collection. EMS agencies in Seoul, Tokyo, Osaka, Taipei, and Singapore utilize established electronic patient care record systems for all EMS cases [20]. This allows a more consistent and sustainable data collection for multiple purposes, including for performance analysis. Unfortunately, the EMS agencies in Bangkok and Kuala Lumpur do not use similar electronic patient care record systems. Most patient care records are manual, and data collection is not standardized and non-uniform. Interestingly, the obvious difference in the structure of EMS between the developed and developing countries in Asia lies with the EMS providers and their location. The cities in the more developed countries utilize the fire department and their personnel as the providers whereas the cities in the developing countries prefer hospital-based EMS provision. Similarly, the training hours for the EMS providers for each city differ significantly, partly due to different training legislation and requirements. The ratio of the number of ambulance to the number of providers varies from 2.16 in Bangkok to 0.05 in Taipei. Such data is unavailable for Kuala Lumpur. Mean response time for out-of-hospital cardiac arrest (OHCA) is within 10 min for all the cities in the developed countries. The response time is much longer in Kuala Lumpur and unavailable for Bangkok. Singapore reported the highest pre-hospital defibrillation rate compared to other cities but unfortunately, the survival to discharge is fairly low. No such data is available for Bangkok and Kuala Lumpur.

There are many challenges in reporting the core measures of EMS performance. Variability in data storage and core measure definitions is very common. Not all countries use a systematic database registry system, especially in the developing countries. Not all data systems utilized similar data dictionaries. This resulted in disparity in the way measurements were derived. Some EMS agencies followed the measure exactly, while others had interpreted the measure more loosely. Many local EMS systems use paper pre-hospital patient care records while others use the electronic format. Abstracting information from paper forms is difficult, time-consuming, and not necessarily accurate [21]. In contrast, some software systems have a high degree of technological sophistication that forces users to complete forms before closing the record [22]. Moreover, without training in the specific core measures, users may not have understood the criticality of completing each data point. One of the clear challenges identified was the difficulty and inability by EMS agencies to obtain universal hospital outcome data on every ambulance transport. This was evidenced by the low response rate for specific cardiac arrest outcome measures. These measures relied upon the hospital to report survival to emergency department discharge and survival to hospital discharge. Another significant limitation of reporting EMS information is related to the nature of the “tiered” EMS system that is present in cities like Kuala Lumpur. Because there are EMS first responders and separate ambulance transport units that arrive at a later time, often, two records are initiated for each patient. This inability to aggregate first responder data with transport provider data could lead to a conclusion that care was not provided, when in fact, it may have been provided to the patient by a different provider.

#### Recommendation

A few efforts should be considered in order to reduce the limitations in obtaining universal and uniform EMS performance measures in the Asian countries. The establishment of the PAROS in the Asian region in 2010 has significantly changed the landscape of networking among EMS experts in the region. The PAROS is a collaborative research group formed by dedicated pre-hospital emergency care (PEC) providers conducting PEC research in the Asia-Pacific region. The initial step involved the data collection pertaining to the OHCA within the Asian region. Before the study was conducted, basic data on the EMS components were obtained from the involved countries and cities, namely, the general structure, process, and outcome of the EMS system. The regional networking has created standardized definitions across the PAROS network by adopting a consensual common taxonomy and data collection methodology. This will allow consideration in an attempt to create uniform EMS measures in the region and to identify common available core measures from each country or cities. The uniform core measures will create a more meaningful EMS practice comparison and benchmarking in the region. This is a very important step to a very long journey in creating a sustainable unique, low-cost, self-funded model of a collaborative research and education network in EMS. No doubt, there will be a lot more hurdles to be faced but with perseverance and commitment this effort will succeed.

### Conclusion

EMS is an integral part of every community’s total health care delivery system. Consistent evaluation of clinical and response performance indicators are crucial components in ensuring that first response services are operating at peak efficiency. To achieve this, continuous quality improvement (QI) practices must become an essential and seamless part of normal EMS routines. Unfortunately at the present time, there is an obvious significant difference in the EMS practice of reporting in the Asian region. This creates difficulty for EMS performance index comparison and benchmarking. Data systems that are robust and agile, with the ability to report clinical indicators and performance measures, are a key tool in quality improvement activities. Hopefully, in the future, collaborative efforts such as the PAROS networking group will further enhance the standardization in EMS performance reporting across the region.

## Abbreviations

EMS: emergency medical services
PAROS: Pan-Asian Resuscitation Outcome Study
EMSPM: Emergency Medical Services Performance Measurements
IOM: Institute of Medicine
NHTSA: National Highway and Traffic Safety Act
OHCA: out-of-hospital cardiac arrest
PEC: pre-hospital emergency care
